# Bidirectional electroactive microbial biofilms and the role of biogenic sulfur in charge storage and release

**DOI:** 10.1016/j.isci.2021.102822

**Published:** 2021-07-07

**Authors:** Paniz Izadi, Marten Niklas Gey, Nicolas Schlüter, Uwe Schröder

**Affiliations:** 1 Institute of Environmental and Sustainable Chemistry, Technische Universität Braunschweig, Hagenring 30, 38106 Braunschweig, Germany

**Keywords:** Applied Microbiology, Microbiofilms, Energy Systems, Energy Storage

## Abstract

The formation of combined electrogenic/electrotrophic biofilms from marine sediments for the development of microbial energy storage systems was studied. Sediment samples from the German coasts of the Baltic and the North Sea were used as inocula for biofilm formation. Anodic biofilm cultivation was applied for a fast and reproducible biofilm formation. North-Sea- and Baltic-Sea-derived biofilms yielded comparable anodic current densities of about 7.2 A m^−2^. The anodic cultivation was followed by a potential reversal regime, transitioning the electrode potential from 0.2 V to −0.8 V every 2 h to switch between anodic and cathodic conditions. The charge-discharge behavior was studied, revealing an electrochemical conversion of biogenic elemental sulfur as major charge-discharge mechanism. The microbial sequencing revealed strong differences between North- and Baltic-Sea-derived biofilms; however with a large number of known sulfur-converting and electrochemically active bacteria in both biofilms.

## Highlights

• Bidirectional electroactive biofilms are cultivated from marine sediments
• Cultivation is based on anodic growth followed by periodic potential reversal
• Combined electrogenic and electrotrophic activity is shown
• Biogenic, elemental sulfur plays a key role in charge storage and release

## Graphical abstract

**Figure:**
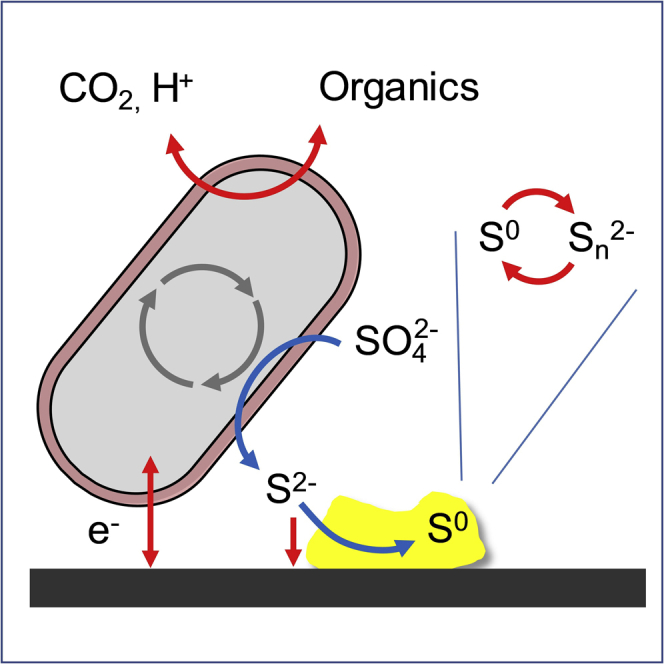

## Introduction

In the search for sustainable methods for electricity generation and CO_2_ reduction, microbial electrochemical technologies (METs) have attracted significant attention. METs employ microorganisms as biocatalysts in anodic, cathodic, or both compartments (Schröder et al., 2015; Izadi and Yu, 2020). Biocatalysts in anodic compartments are known for catalyzing organic degradation and electricity generation (microbial fuel cell [MFC] platform), while reductive reactions such as CO_2_ reduction (microbial electrosynthesis platform) can be catalyzed by cathodic biocatalysts. Regardless of the platforms, the basis for the above technologies lies in the interaction of microorganisms and electrodes, also known as extracellular electron transfer (EET) (Ter Heijne et al., 2020). Depending on the direction of the electrons flow, we can distinguish between outward and inward EET, where outward EET is when electrons flow from the microbial cells to electrodes (anodic current; often referred to as electrogenesis), whereas inward EET describes electron flow from an electrode to the microbial cells (cathodic current; often referred to electrotrophy) (Kumar et al., 2017). Biocatalysts capable of bidirectional EET interaction can potentially be exploited for energy storage and release (Molenaar et al., 2016; Malvankar et al., 2012). Thus, a single electrochemically active biofilm can switch between energy storage and energy release, which advances the bioelectrochemical systems' applications. This application can be in particular applied for locations with harsh conditions of high salinity or alkalinity for instance, such as lakes and marine sediments (Yates et al., 2017). Despite the recent studies on microbial EET, information on the mechanisms involved in bidirectional EET is still scarce – also owing to the limited knowledge on the identity of the microorganisms with bidirectional EET capability. This is more particular for inward EET, as the interaction mechanisms involved between cathodes and microorganisms have not been fully understood yet (Jiang et al., 2019). So far, few commonly known electroactive bacteria such as *Geobacter* and *Shewanella* have been shown to be able to catalyze both anodic and cathodic reactions (Choi and Sang, 2016; Chu et al., 2021), with cytochromes being main EET components. Thereby, the bidirectional EET is based on a reversible conduit and reversible interfacial EET pathways (Jiang and Zeng, 2019). Different *Clostridium* spp. have been shown to serve as cathode catalysts for the production of organic compounds from CO_2_ (Izadi et al., 2021); however, they have also been found in bioanodes for electricity generation (Koch and Harnisch, 2016). Further studies are required to identify more microorganisms able to perform as both anodic and cathodic biocatalysts and to clarify the mechanisms involved.

As illustrated by Yates et al., the cultivation of electrochemically active biofilms that possess bidirectional metabolic activities can be achieved via autotrophic cultivation and under application of a polarization reversal regime (Yates et al., 2017). A major experimental hurdle of the underlying procedure is an extremely long lag time that can require more than one hundred days of cultivation before significant current flow is observed. Since the original inoculum of Yates et al. was based on a microbial consortium from the anode of an active sediment fuel cell, it seems obvious that a primary selection of all relevant microbial species took already place during the heterotrophic (anodic) MFC operation.

Another strategy suggested for the development of bidirectional biofilms was a one-time polarity reversal in which an anodic biofilm was first enriched from the mixed population of microorganisms under anodic conditions, followed by switching the electrode potential and forcing the bioanode to function as a biocathode (Pisciotta et al., 2012; Lin et al., 2021). Enrichment of nondesired microbial communities such as methanogens seemed to be one of the challenges of this strategy deteriorating the system efficiency.

In a recent study on switchable biofilms, the importance of the substrate used for the development of the bioanode on the inverted electrotrophic activity of the biofilm was pointed out (Hartline and Call, 2016). Although the identity of the microorganisms was not clarified, the authors suggested that bianodes enriched by formate showed more significant cathodic activities after switching the polarity (Hartline and Call, 2016).

In this study, we investigated the formation of bidirectional electrochemically active biofilms from marine sediment samples from the Baltic Sea and from the North Sea for the aim of a reversible bioelectrochemical energy storage. It was one of the goals to establish a fast and reliable biofilm cultivation strategy. We therefore combined approaches described above by utilizing a primary heterotrophic (anodic) biofilm formation step using formate, acetate, and butyrate as substrates, followed by a periodic potential reversal. The electrochemical and biological properties of the resulting biofilms were studied and compared between North-Sea- and Baltic-Sea-based biofilms in order to elucidate the potential mechanisms involved in the bidirectional electron transfer.

## Results and discussion

### Biofilm cultivation

Using the anodic biofilm cultivation described in the experimental part, for all triplicates of the North reactors, current generation began approximately 5 days after inoculation, reaching the peak current of 7.2 ± 2.4 A m^−2^ on day 10 (Figure S1A). For the Baltic reactors, reproducible current generation was observed only after about 18 days, indicating a longer time necessary for the microbial community to adapt to the environmental conditions (Figures S1B and 1, Inset). One possible reason for the considerably longer lag period is the adaptation to the salt contents of the growth medium. Thus, with about 2.3%, the salinity of the growth medium is much closer to the salinity of the North Sea (about 3.5%) than of the Baltic Sea (0.9%).

**Figure 1:**
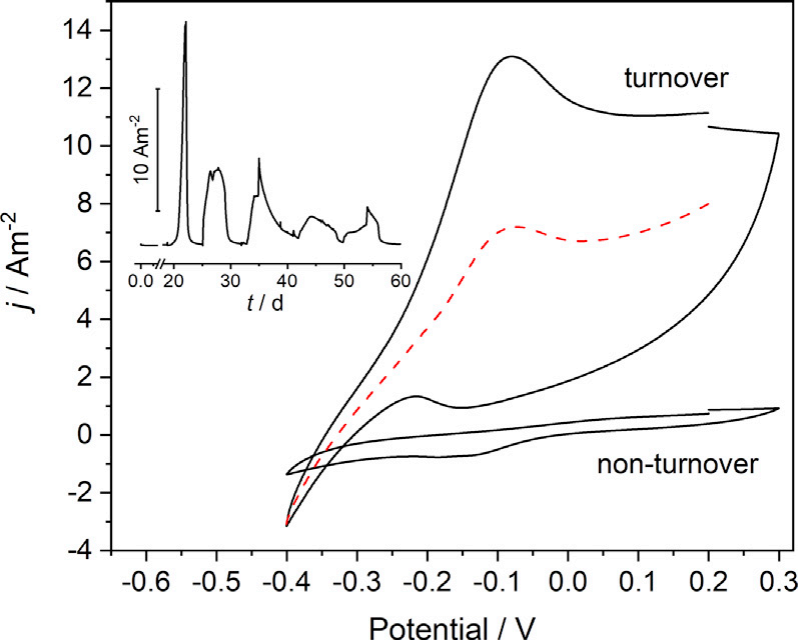
Electrochemical characteristics of the anodic biofilm cultivation Inset Figure: Exemplary chronoamperometric curve of the anodic biofilm cultivation of a Baltic biofilm (See Figures S1A and S1B for all North and Baltic cultivation data). Main Figure: Cyclic voltammograms of a Baltic biofilm under turnover and non-turnover conditions (Scan rate 1 mV s^−1^).The red, dashed line represents an averaged voltammetric curve (*j* = (*j*_forward_ + *j*_backwards_)/2).

Despite the longer lag phase of the Baltic biofilms, they reached current densities similar to the North biofilms. In all the batch cycles, formate was depleted by the end of the respective cycles, showing the preferential consumption of this compound (See Figures S3 and S4). Acetate and butyrate were increasingly consumed in the course of the cultivation; whereby especially during the first batch cycles, a buildup of butyrate was observed, which may stem from organic compounds in the original sediment inoculum. After 30 to 40 days of reproducible anodic current generation, we decreased the total substrate concentration in the fresh medium from 2 g L^−1^ COD to 1 g L^−1^ COD to provide conditions more similar to marine sample conditions (see arrow in the chronoamperometric growth curve in Figure 1).

The cyclic voltammograms obtained after the anodic biofilm cultivation are relatively ill-defined. On the one hand, the turnover voltammetry clearly indicates electrocatalytic properties in the biofilms. Thus, the currents of the turnover voltammograms (see Figure 1 for the Baltic biofilm and Figure S2 for the North Biofilm) show a typical shift of the anodic region toward oxidizing values. As illustrated by the red curve in Figure 1, an averaging of the forward and the backwards CV scan yields a curve with an approximately sigmoidal shape with a half-wave potential of approximately −0.25 V. However, a strong hysteresis of the forward and the backward scan of the pristine CV at potentials more positive than −0.2 V, as well as the occurring peaks at −0.08V (oxidation peak) and −0.15 V (reduction peak) indicate an overlapping of the catalytic curve with a noncatalytic electrochemical process. The overlapping becomes even more obvious for the North biofilm voltammetry (Figure S2) in which the anodic current density of the turnover voltammogram of 35 A m^−2^ clearly exceeds the stationary current densities achieved at constant potential (7.2 A m^−2^). For both biofilm types, this superimposition makes a more quantitative analysis of the bioelectrocatalytic process impossible. The origin of the noncatalytic process, however, will be discussed later in this manuscript.

**Figure 2:**
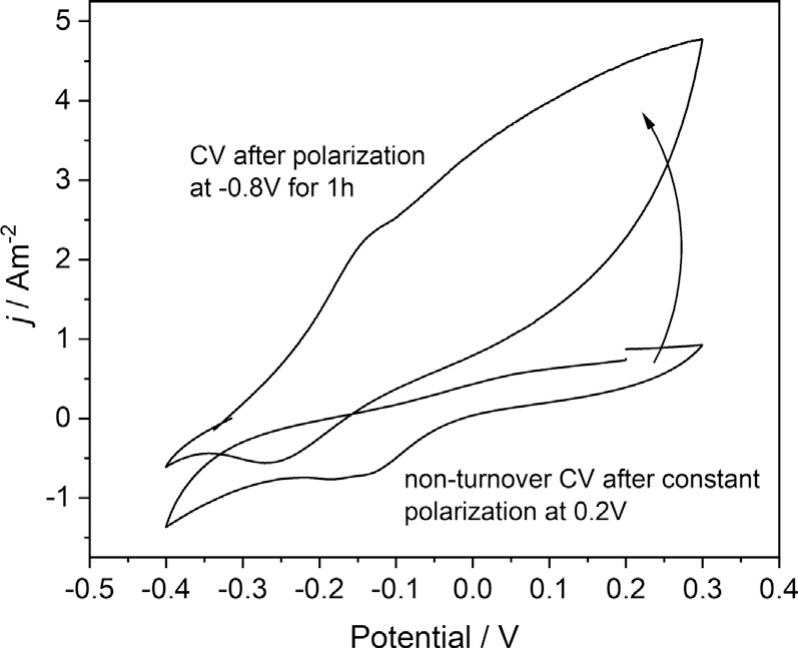
Impact of a cathodic polarization on the non-turnover biofilm voltammetry The figure shows cyclic voltammograms of a Baltic biofilm under nonturnover conditions, before and after polarization at a potential of −0.8V for one hour (Scan rate 1 mV s^−1^).

When polarizing the biofilm under nonturnover conditions – i.e., in the absence of an organic substrate, yet in the presence of bicarbonate – at a negative potential for a certain period, the subsequently recorded voltammograms reveal the return of an oxidative electrocatalytic process (Figures 2 and S5). This behavior indicates an electrotrophic conversion of inorganic carbon into an organic compound during the negative polarization and the subsequent oxidative degradation of the formed compound.

This hypothesis was later confirmed by electrolysis experiments in which the biofilms were exposed to constantly applied −0.8 V electrode potential for 5 days (see chronoamperometric curves in Figure S6). After 5 days, not only H_2_ was found in the headspaces of all reactors but also formate (*ca.* 0.97 mM) and acetate (*ca.* 1.17 mM) production was observed in the solutions of Baltic reactors representing respective *ca.* 15.9% and 76.8% of the total cathodic current – which confirmed microbial electrosynthesis in these reactors. In the North reactors, hydrogen gas was the sole product.

### Periodic potential reversal for bidirectional electron transfer

**Figure 3:**
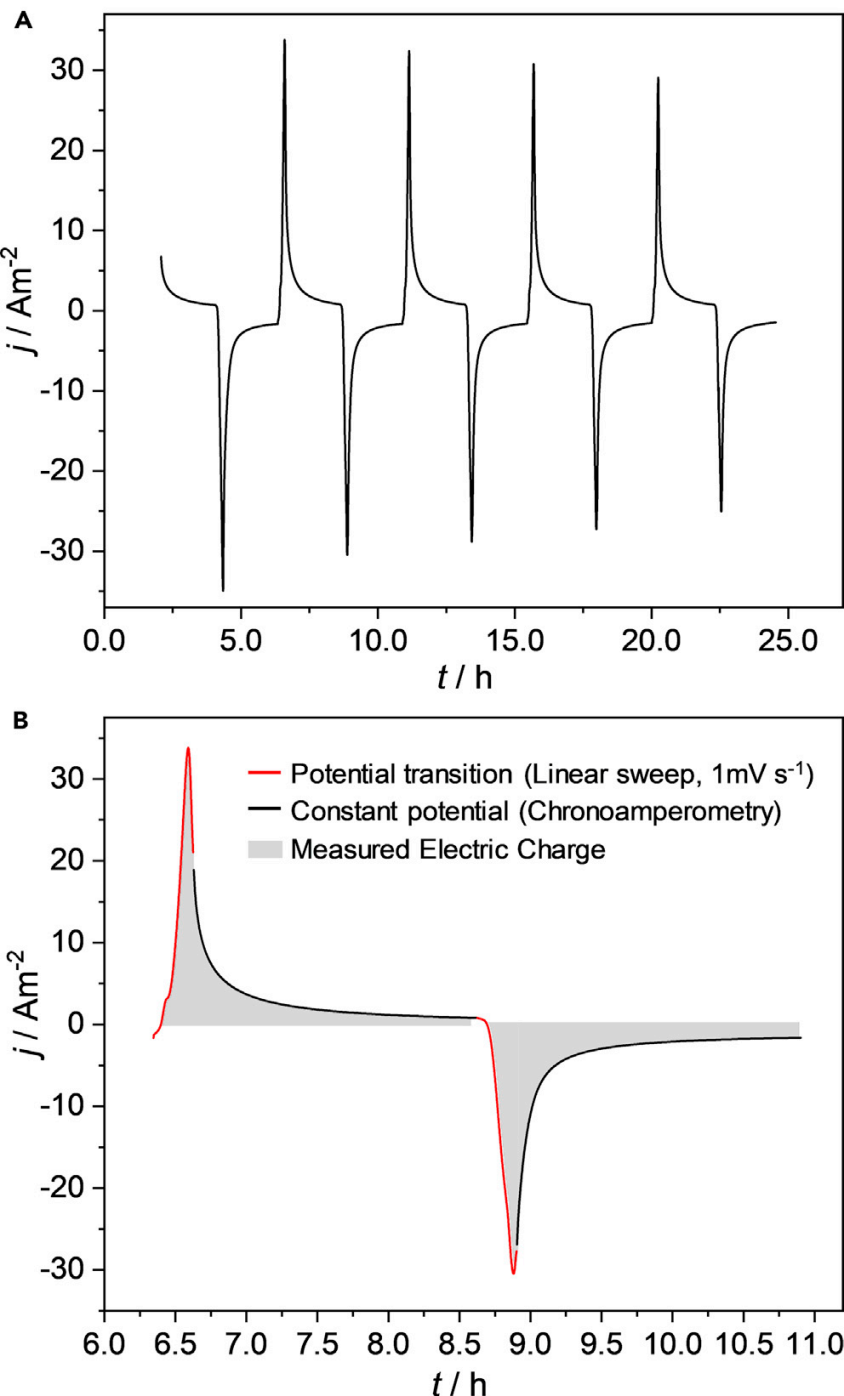
Electrochemical biofilm performance during a periodic potential reversal (A and B)(A) Oxidative and reductive current densities generated during a periodic potential reversal of a Baltic biofilm (B) Time segment illustrating of (A) illustrating one potential reversal loop. The cultivation medium contained solely inorganic carbon (HCO_3_^-^).

When the development of anodic biofilms at the electrodes was assured (48 days for the North biofilms and 60 days for the Baltic biofilms), we applied a periodic potential reversal regime by transitioning the poised electrode potential from 0.2 V to −0.8 V every 2 h (see experimental part for details), in order to study the bidirectional biofilm behavior. Figure 3 depicts the resulting oxidative and reductive currents for five exemplary potential reversal loops. The charge-discharge curves show maximum current densities of around 30 A m^−2^ for both oxidation and reduction. These peak currents were reached during the potential ramp that was used for switching between oxidation and reduction potential (see Figure 3B). After reaching the respective currents peaks, the oxidation and reduction currents show a strongly transient behavior, with an at least exponential decay during the fixed potential periods. A significant contribution of capacitive currents (double layer charging/discharging) can be excluded, since the transition between oxidation and reduction potential occurs via a slow potential sweep of 1 mV s^−1^ (see Figure 3B). Instead, the behavior can be described as pseudocapacitive – based on an exhaustive conversion of surface-confined electrochemically active species. Pseudocapacitive behavior has been described for e.g., *Geobacter sulfurreducens* biofilms and was ascribed to the charging/discharging of the cytochrome pool within the *Geobacter* biofilm (Malvankar et al., 2012).

**Figure 4:**
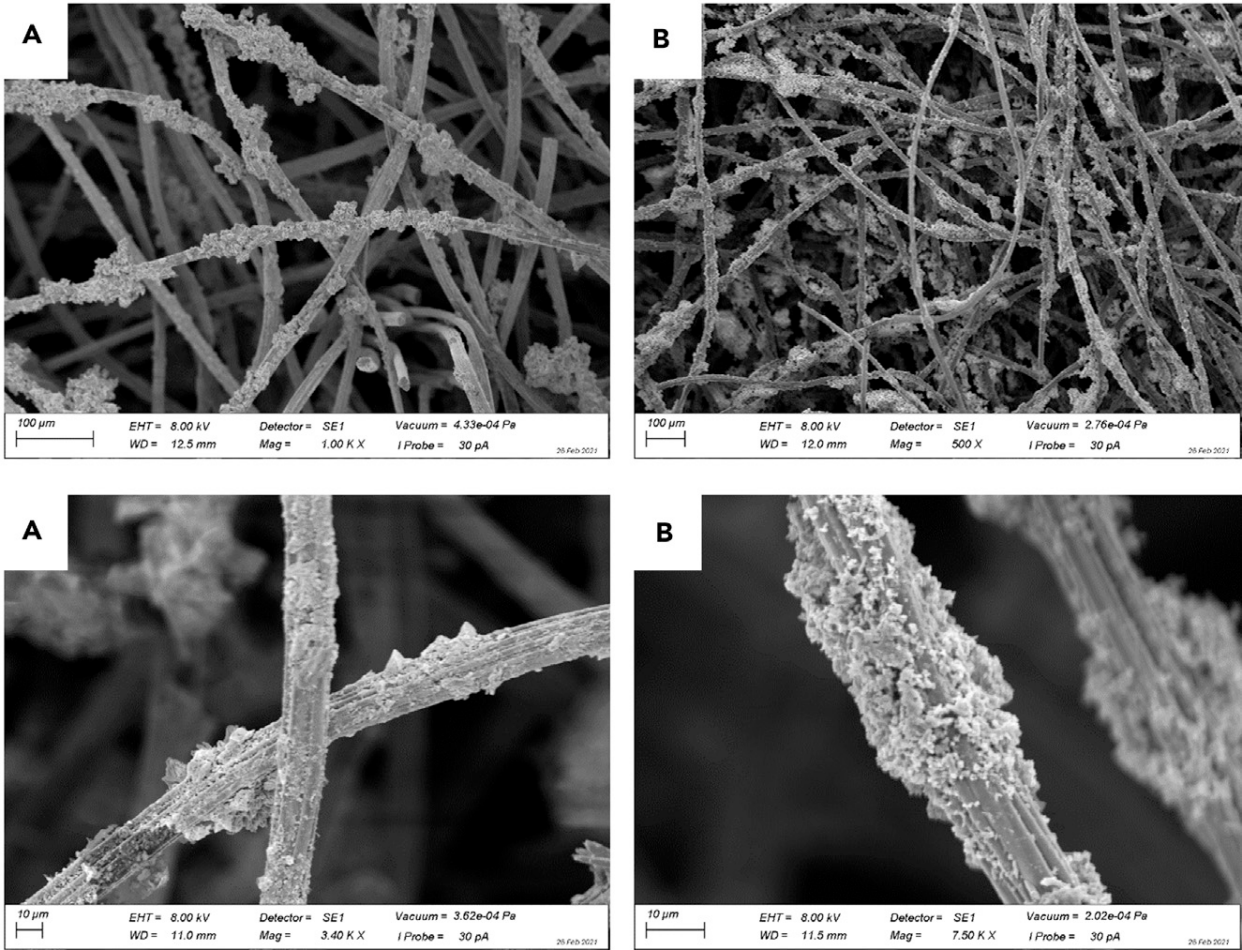
Scanning electron microscopy images of cultivated biofilm electrode (A and B) (A) North and (B) Baltic biofilm electrodes; The images were taken at the end of the experimental period.

A charge balance graph for all North and Baltic biofilms is shown in Figure S9. By integrating the current density data depicted in Figure 3B, a charge density of 31 kC m^−2^ (oxidation) and 37 kC m^−2^ (reduction) can be derived. Taking the comparatively low surface coverage of the carbon electrode fibers (See Figure 4) into account – which is lower than that of typical anodic biofilms – this high faradaic current/charge density is surprising.

### Bidirectional biofilm microbial communities

**Figure 5:**
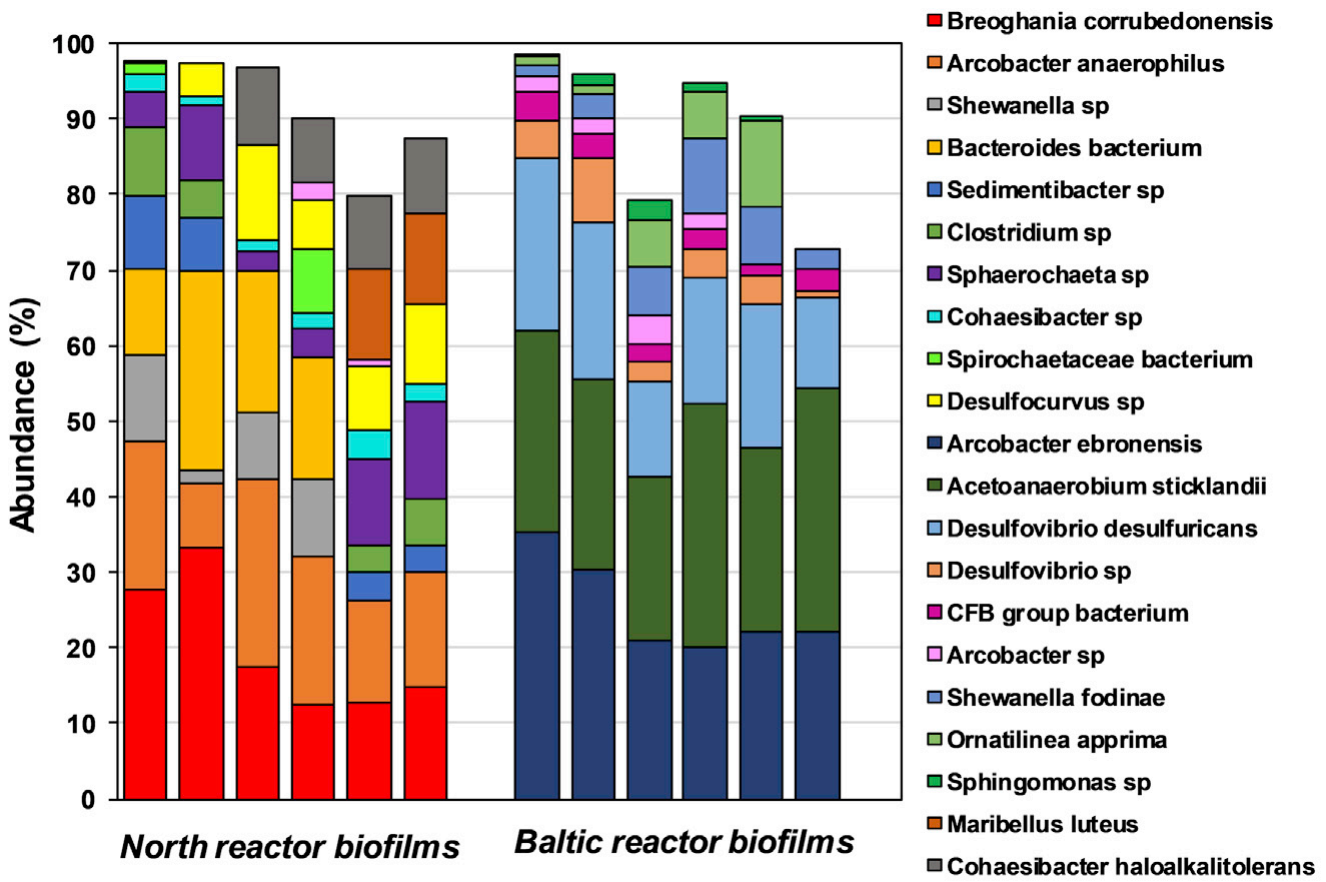
Abundance of the microbial communities at the genus level The analysis of the North and Baltic biofilms was performed after completing the periodic potential reversal experiment. Each group of six stacked bars indicates microbial communities from duplicate biofilm samples from each of three replicate BES reactors.

The microbial communities in the biofilms were analyzed to clarify the identity of the enriched microorganisms (Figure 5). The most dominant microorganisms in the Baltic biofilms were *Arcobacter ebronesis* and *Acetoanaerobium sticklandii*, representing 25.2 ± 6.2% and 27.0 ± 4.3% of the microbial cells in the biofilms, respectively. In the North biofilms, *Arcobacter anaerophilus* comprised 16.9 ± 5.7% of the biofilms' microbial communities. *Arcobacter* spp. are known sulfide oxidizing marine bacteria and the *A. ebronesis* sequence detected in the Baltic samples showed high similarity to *Desulfovibrio desulfuricans* (see phylogenetic tree in Figure S8). *Arcobacter* spp. have been reported to be involved in the anodic reaction of a microbial fuel cell inoculated with marine planktons (Reimers et al., 2007). Additionally, the sulfide oxidizing marine bacteria of *Arcobacter* spp. have been reported as autotrophic bacteria capable of carbon fixation, indicating their capability in a potential cathodic CO_2_ reduction (Wirsen et al., 2002). *A. sticklandii* (also known as as *Clostridium sticklandii* [Galperin et al., 2016]), enriched in the North biofilms, has been found abundantly at an MFC anode operating at a low temperature of 10°C (Dai et al., 2020). In general, *Clostridium* spp. are known for a cathodic production of longer-chain carboxylic acids (Izadi et al., 2021). Similarly, *C. sticklandii* has also been found in the microbial communities enriched during glucose electrofermentation (Toledo-Alarcón et al., 2021). In addition to *C. sticklandii* in the North biofilms, species of *Clostridium* were also found in the Baltic biofilms, which could justify acetate and formate production during the cathodic condition in both reactors. In the North biofilms, *Breoghania corrubedonensis* was another abundantly enriched microorganism dominating 19.7 ± 8.7% of the biofilm's microbial communities.

This species has not been detected in previous BES studies yet, however, it showed high similarity to *D. desulfuricans* (Figure S8). Species of the *Desulfovibrio* genus, found in both Baltic and North biofilms (Figure 5), are known sulfate reducing bacteria able to produce H_2_ in cathodic compartments. Previous study on H_2_ production using *Desulfovibrio* spp. showed a high coulombic efficiency of nearly 100%. In that study, bacterial cells were grown using organic compounds such as lactate or H_2_ as electron donors and sulfate as electron acceptor (Aulenta et al., 2012). However, after transferring the bacterial cells to the cathodic compartments of a bioelectrochemical reactor and removing sulfate from the medium, the bacteria were able to use protons as electron acceptors instead of sulfate, leading to H_2_ production 20 times higher than in the presence of sulfate. The authors emphasized the activity of hydrogenase at the cathode surface and its involvement in catalyzing the H_2_ production (Aulenta et al., 2012). The ability of *D. desulfuricans* for anodic electricity generation and the role of cytochrome *c* as EET component has also been discussed (Kang et al., 2014). These studies in addition to our results indicate the potential capability of bidirectional EET of *Desulfovibrio* spp.

*Bacteroides* bacterium was found in North biofilms comprising 18.0 ± 6.1% of the microbial communities. *Bacteroides* has been found as one of the most abundant genera in anodic biofilm (Ishii et al., 2014; Lakaniemi et al., 2012) and has been proposed to be involved in anodic reactions. It has also been found as one of the dominant genera in H_2_-producing cathodic biofilms (Croese et al., 2011). Additionally, *Shewanella* spp. were found in both Baltic and North biofilms. *Shewanella* are known to be able to function as both bioanode and biocathode, with an Mtr pathway as well as a flavin based electron shuttling being involved in their extracellular electron transfer (Jiang and Zeng, 2019). *Shewanella* spp. have primarily been studied for anodic reactions; however, recent studies have shown that *Shewanella* spp. are also capable of performing cathodic reactions (Rowe et al., 2018; Philips et al., 2018). Thus, *Shewanella fodinae*, which was also found in the samples of this study, are able to consume carbonate as an inorganic source using electrons derived from a cathodically polarized electrode (Philips et al., 2018). The results of community analysis confirm the development of biofilms with potential ability of bidirectional extracellular electron transfer.

### The occurrence of biogenic elemental sulfur at the electrode surface

**Figure 6:**
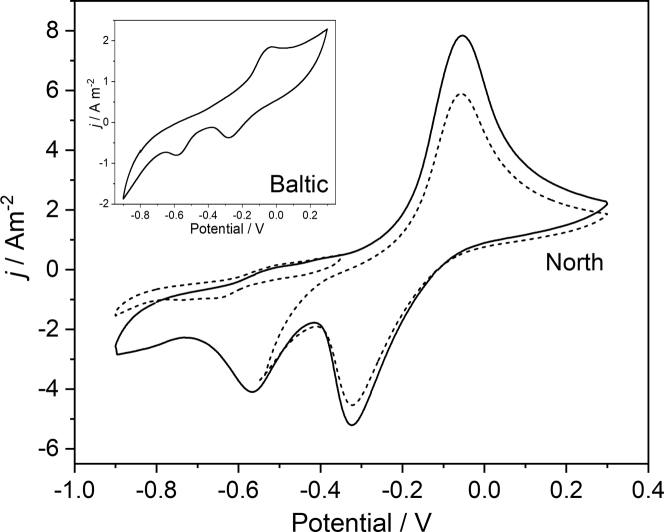
Voltammetric biofilm behavior after the potential reversal experiment The figure depicts the cyclic voltammograms of a North biofilm (Main figure) and of a Baltic biofilm (Inset) were recorded after the potential reversal experiment, and at a scan rate = 1 mV s^−1^. The cultivation medium contained solely inorganic carbon (HCO_3_^-^). The dotted lines indicate voltammograms recorded in narrower potential windows.

The voltammograms recorded after periodic potential reversal (Figure 6) are different in their appearance than the voltammograms shown in Figure 1. Apart from a potential impact of the potential reversal experiments, it has to be noted that (i) the voltammograms shown in Figure 6 were recorded over an extended cathodic range and (ii) the medium did not contain an organic carbon source, but solely inorganic carbon (CO_2_/bicarbonate). It is astonishing to see that despite the strong differences in the composition of the microbial communities of North and Baltic biofilms, the voltammetric behavior of the biofilms (Figure 6), recorded after the potential reversal experiments, shows a high degree of similarity. Thus, the main features of the voltammograms are single oxidation peak (at a potential around −0.05 V) and two reduction peaks (around −0.32 V and −0.56 V). As the dotted curves in Figure 6 illustrate, the occurrence of the negative reduction peak is bound to the main oxidation process. Cyclic voltammograms recorded within a narrower, less positive oxidation range did not show the occurrence of this reduction peak. Voltammograms recorded only in a positive potential window yielded a decrease in the height of the oxidation peak. This is in accordance with results of a charge integration of the main voltammograms, which shows that the integrated charge underneath both reduction peaks corresponds to the charge underneath the oxidation peak.

**Figure 7:**
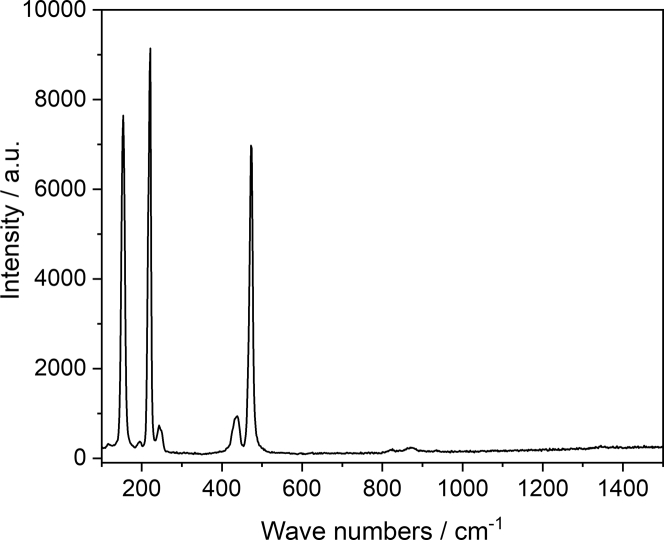
Raman spectrum of an exemplary biofilm electrode The figure depicts a Raman spectrum of an exemplary North biofilm recorded after the periodic potential reversal experiment. The figure proves the presence of elemental sulfur at the biofilm electrode.

Based on the similarity of the Baltic and North biofilm voltammograms, one could suspect that either one of the dominating microbial species that are present in both – North and Baltic biofilms – are responsible for this similarity, or the involvement of a chemical compound that is formed at the electrode surfaces. In order to answer this question, we used Raman spectroscopy to analyze the chemical composition of the biofilms and of the electrode surface. As depicted in Figure 7, with characteristic peaks at 155, 223, and 478 cm^−1^ (Himmel et al., 2009), Raman spectroscopy revealed the presence of elemental sulfur at the electrode surface.

The presence of sulfur was surprising, since the cultivation medium did not contain any sulfates (see experimental part). However, as depicted in the Table of the Star Method section, the original sediment, which served as inoculum, contained relatively high levels of sulfate – which can be considered as the primary source for sulfur compounds in the reactors. Sulfate ions can serve as terminal electron acceptor for sulfur-reducing bacteria abundant in all biofilms – either by using an organic electron donor during the primary cultivation step or via electron uptake from the electrode during cathodic polarization (Agostino and Rosenbaum, 2018). Thereby, the product of a complete sulfate reduction is sulfide (under neutral pH conditions mainly HS^−^). Under anodic conditions, sulfide ions can be reoxidized, whereby depending on the electrode material, reoxidation can be either complete and can be utilized for electricity generation (Habermann and Pommer, 1991) or can lead to the formation of elemental sulfur, which has previously been reported to scale anodes in microbial fuel cells due to its electronically insulating properties (Rabaey et al., 2006). In the light of this finding, the SEM images depicted in Figure 4, may partly show sulfur deposits on the electrode surface (see for comparison Figure 2 in [Dutta et al., 2009]).

### The role of elemental sulfur in electrode charging-discharging

**Figure 8:**
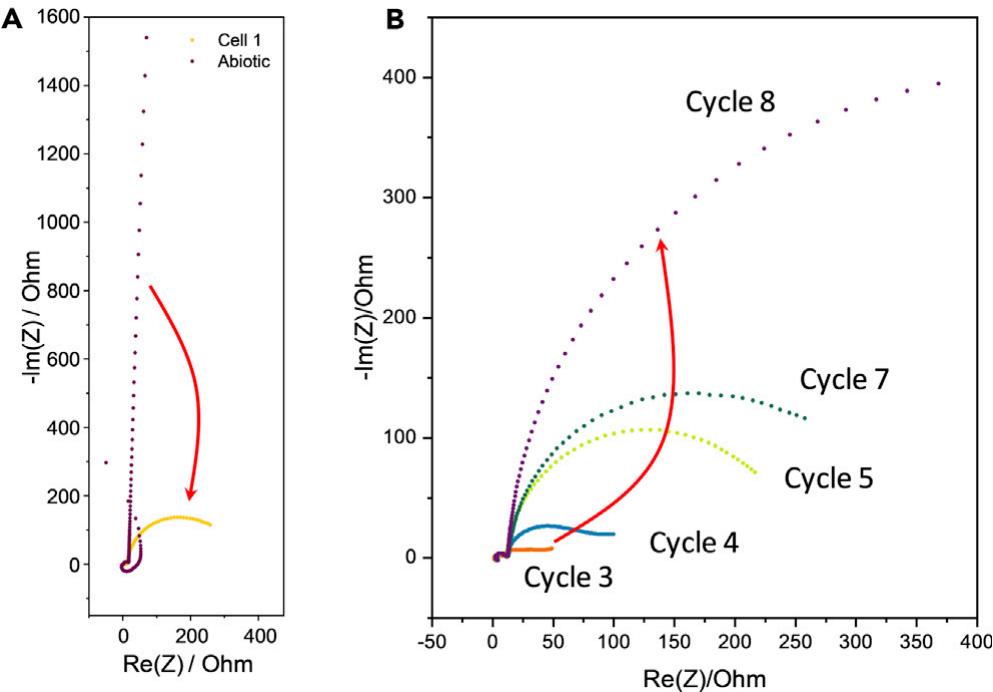
Electrochemical impedance spectra at different stages of biofilm formation Exemplary impedance spectra (Nyquist plots) (A) of a graphite electrode in the cultivation medium before and after anodic biofilm formation; (B) of a biofilm electrode during the course of the anodic cultivation (North biofilm; reactor 1). The spectra were recorded at the respective current maxima of the cultivation semibatch cycles, at a polarization potential of +0.2V.

The formation of sulfur at the electrode surface may explain a peculiar behavior that we observed using electrochemical impedance spectroscopy (EIS) during biofilm cultivation. On the one hand, EIS data reveal the transition of the electrode behavior from blocking (purely capacitive) to charge-transfer upon microbial electrode colonization. This transition is visible in the impedance curve of the Nyquist plot shown in Figure 8 A: Beside a small semicircle that is due to the contact resistance of the graphite electrode (and that remains unaltered for all cycles), the abiotic control is dominated by a straight line with a phase shift angle of nearly 90° representative for a purely capacitive behavior without any charge transfer processes. With colonization of the electrode, a second semicircle occurs that combines faradaic and capacitive behavior – typical for charge-transfer processes. This is an expected outcome that reflects the enrichment of electrochemically (and electrocatalytically) active bacteria at the electrode surface, allowing faradaic currents to flow based on their electrogenic activity. On the other hand, however, the charge transfer resistance increases again in the course of the colonization process (from 15 Ohms [cycle 3] to 419 Ohms [cycle 8]) – as visible in the increasing diameter of the lower frequency semicircles in the respective Nyquist plots (Figure 8B). A more detailed and representative analysis of the electrochemical impedance data and details about the equivalent circuit fitting, as well as exemplary EIS data from Baltic biofilm electrodes, are shown in the supplementary information (Figures S11–S16). At this point, it has to be noted that due to a strong time variance of the spectra, especially (visible in a Kramer-Kronig analysis – data not shown, as well as in a “tailing” of the CT semicircles), a quantitative analysis of the impedance data of the Baltic biofilms was not undertaken.

Under the conditions of the experiment, i.e., at a constant polarization of 0.2 V, sulfur is electrochemically inactive and acts as an electronic insulator. It is thus reasonable to assume that the strong increase in the charge transfer resistance during the cultivation is caused by the deposition of elemental sulfur at the electrode surface, impeding the microbial extracellular electron transfer. The increasing charge transfer resistance may also deliver an explanation for the declining current density level during the biofilm cultivation (Figure S1). It should, however, also be noted that the amount of substrate was reduced by 50% during the cultivation (see arrows in Figure S1), which is also likely to contribute to the reducing current density level.

Is the sulfur deposit thus detrimental for an envisioned bioelectrochemical charge storage and release application? On the contrary, we thus hypothesize that the charge-discharge currents shown in Figure 3 in main parts can be attributed to the reduction and reoxidation of a sulfur-sulfide/polysulfide system. It is thus well known that sulfur can be electrochemically reduced to sulfide – this was utilized by Dutta et al. for the removal of sulfur from the anode of a domestic wastewater MFC (Dutta et al., 2009). Moreover, the sulfur reduction is also the functional basis of sulfur batteries (Kang et al., 2016). It is thereby intriguing that, although sulfur batteries utilize organic solvents, typical cyclic voltammograms of sulfur electrodes possess the same peak characteristics as the voltammogram depicted in Figure 6, comprising one oxidation process and two reduction peaks (Su and Manthiram, 2012; Wang and Zhang, 2020), with the separation between the two reduction peaks being virtually identical with the peak separation found in our study (See Figure S9). Thereby, the two reduction peaks have been ascribed to the reduction of elemental sulfur (S^0^) to polysulfide (S_n_^2−^) at relatively positive potential (and with only low peak separation to the oxidation peak) and the reduction of the polysulfide to sulfide (S^2−^) at relatively negative potentials (large peak separation).

**Figure 9:**
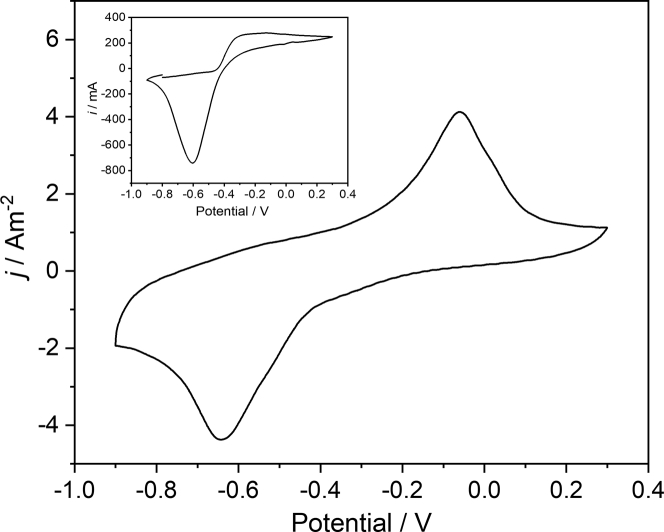
Cyclic voltammetry of a biofilm electrode and of an abiotic sulfur-modified electrode Main figure: Cyclic voltammogram of a North biofilm recorded after the periodic potential reversal experiment (Scan rate = 1 mV s^−1^). Different to the CV shown in Figure 6, the cultivation medium did not contain bicarbonate. Inset figure: Cyclic voltammogram of a (abiotic) sulfur-graphite composite electrode, recorded in sterile growth medium and in the presence of 5 mM sulfide (Scan rate 10 mV s^−1^).

In order to prove the above hypotheses, we recorded voltammograms of an abiotic graphite-sulfur composite electrode in a sterile bacterial buffer solution. As depicted in Figure 9 (inset) and as supported by literature, the voltammetry of the sulfur-sulfide system in aqueous environments often shows only one reduction peak, presumably representing the complete reduction to sulfide (Thompson, 1988). The peak potentials for the abiotic sulfur electrode was measured with *E*_p,ox_ = −0.13 V and *E*_p,red_ = −0.13 V – which is in good agreement with the biotic system (Figures 6 and 9, main figure). Interestingly, under some conditions – like the absence of bicarbonate in the bacterial growth medium – also the biotic electrodes (see Figure 9, Main figure) showed this appearance. This illustrates that the mechanisms of the sulfur reduction are strongly dependent on the environmental conditions. A potential involvement of (sulfur-oxidizing) bacteria cannot be excluded.

For a low-loss energy storage and release, large peak separations as shown in Figure 9 should clearly be avoided and instead, the redox cycling should be confined on the electrochemically, more positive subsystem (see dashed CV curve in Figure 6). In a follow-up study, we will therefore investigate how this redox system can be explicitly exploited.

### Conclusions

In this study, we have shown that biofilm electrodes capable of bidirectional electron transfer can be enriched from marine sediments from the Baltic Sea and the North Sea via an anodic cultivation strategy, followed by a periodic potential reversal regime. North-Sea- and Baltic-Sea-derived biofilms differ in their specific microbial composition; however, they are, independently of their origin, dominated by bacteria with known electrogenic and electrotrophic properties and by sulfur-reducing and sulfur-oxidizing bacteria.

In heterotrophic (electrogenic) mode, i.e, in the presence of organic compounds, the biofilms are able to degrade organic acids, such as formate, acetate, and butyrate under anodic current generation. In autotrophic (electrotrophic) mode, inorganic carbon is being utilized for the cathodic generation of organic acids, such as formic acid.

Short-term electrode behavior, however, is dominated by the electrochemical conversion of sulfur, which is formed at the electrode surfaces upon oxidation of biogenic sulfide ions. Although elemental sulfur is an insulator and can therefore potentially lead to reduced electron transfer rates, its electrochemical reduction to sulfides (and polysulfides) opens the door for intermitted energy storage and release. Additional work is required to tailor the sulfur deposition and the electrochemical cycling for an optimized electrochemical reversibility.

### Limitations of the study

This study is only the starting point for a more quantitative investigation. For example, a deeper understanding of the amount of sulfur deposited on the electrode as a function of the operational conditions and of the fraction of sulfur actively involved in the charge-discharge process is still missing. Further, the nature of actual microbial extracellular electron transfer remains largely unanswered in this study and requires deeper investigation.

## STAR★Methods

### Key resource table

**Table:** 

REGENT or RESOURCE	SOURCE	IDENTIFIER
**Chemicals and reagents**		

Monopotassium phosphate	Carl Roth	Cat#3904.1
Ammonium chloride	Sigma-Aldrich	Cat#254134
Sodium chloride	Carl Roth	Cat#9265.1
Potassium chloride	Carl Roth	Cat#6781.1
Magnesiumchloride Hexahydrate	Carl Roth	Cat#3532.1
Sodium carbonate	Carl Roth	Cat#8563.1
Calcium chloride dihydrate	Carl Roth	Cat#HN04.2
Sodium bicarbonate	Carl Roth	Cat#6885.1
Sodium selenite pentahydrate	Sigma-Aldrich	Cat#308455
Sodium tungstate dehydrate	Sigma-Aldrich	Cat#72069
D-(+)-Biotin	Sigma-Aldrich	Cat#2031
Folic acid	Sigma-Aldrich	Cat#S579270
Pyrodoxine-HCl	Sigma-Aldrich	Cat#P9755
Thiamine-HCl	Sigma-Aldrich	Cat#T4625
Riboflavin	Carl Roth	Cat#9607.1
Nicotinic acid	Sigma-Aldrich	Cat#N4126
Ca-D-pantothenate	Sigma-Aldrich	Cat#PHR1232
Vitamin B_12_	Sigma-Aldrich	Cat#V2876
4-Aminobenzoic acid	Sigma-Aldrich	Cat#01973
DL-α-lipoic acid	Sigma-Aldrich	Cat#62320
Iron dichloride tetrahydrate	Sigma-Aldrich	Cat#44939
Zinc chloride	Sigma-Aldrich	Cat#208086
Manganese (II) chloride tetrahydrate	Sigma-Aldrich	Cat#221279
Boric acid	Sigma-Aldrich	Cat#B0394
Cobalt (II) chloride hexahydrate	Sigma-Aldrich	Cat#255599
Copper (II) chloride dehydrate	Sigma-Aldrich	Cat#307483
Nickel (II) chloride hexahydrate	Sigma-Aldrich	Cat#654507
Sodium molybdate dehydrate	Sigma-Aldrich	Cat#331058
Hydrochloric acid	Sigma-Aldrich	Cat#320331
Sodium acetate	Carl Roth	Cat#6773.2
Sodium butyrate	Carl Roth	Cat#1441.2
Sodium formate	Carl Roth	Cat#4404.1
Paraformaldehyde	Carl Roth	Cat#0335.1

**Electrode and membrane**		

Silver/Silver chloride electrode	Sensortechnik Meinsberg	Cat#SE10
Cation exchange membrane	Fumatech	Cat#Fumapem-FKE-PP-75

**Software and algorithms**		

R package	RStudio foundation	rstudio:conf 2018 talks - RStudio
Msa	(Bodenhofer et al., 2015)	msa: an R package for multiple sequence alignment | Bioinformatics | Oxford Academic (oup.com)
Ape	(Paradis et al., 2019)	ape.pdf (unipd.it)

### Resource availability

#### Lead contact

Further information and requests for resources and reagents should be directed to and will be fulfilled by the lead contact, Uwe Schröder (uwe.schroeder@tu-braunschweig.de)

#### Data availability

All the sequencing reads have been deposited in the NCBI SRA database under BioProject PRJNA727991.

#### Material availability

This study did not generate new, unique reagents.

### Method details

#### Biofilm cultivation and electrochemical characterization

Dual chamber reactors were used for this study. The chambers were 130 ml glassy compartments separated by a cation exchange membrane (Fumapem-FKE-PP-75, Fumatech, Germany). Graphite felt with a projected surface area of 4 cm^2^, graphite rods with a surface area of 35 cm^2^ and Ag/AgCl electrodes (saturated KCl, Sensortechnik Meinsberg, Germany, 0.197 V vs. SHE) were used as working, counter and reference electrodes, respectively. All electrode potentials provided in this study are reported against the above Ag/AgCl electrode. The electrolyte in the working electrode compartment was 80 ml of sterile growth medium adapted from medium 193b, DSMZ (http://www.dsmz.de). The medium consisted of (per L): 0.2 g KH_2_PO_4_, 0.3 g NH_4_Cl, 7 g NaCl, 0.5 g KCl, 12 g MgCl_2_ x 6 H_2_O, 1.5 g Na_2_CO_3_, 1.5 g CaCl_2_ x 2 H_2_O, 1 mL selenite-tungstate solution according to medium 385 (DSMZ) 5 mL of vitamin solution according to medium 141 (DSMZ) and 1 mL of trace element solution according to medium 320 (DSMZ). Depending on the stage of the experiment, organic or inorganic carbon sources were supplied in the media. Thus, for the heterotrophic biofilm enrichment, 2 or 1 g/L chemical oxygen demand (COD) including 45% formate (28.1 or 56.2 mM), 45% acetate (7.03 or 14.06 mM), and 10% butyrate (0.625 or 1.25 mM) were provided. Formate was selected as it was shown to be able to enrich microorganisms able to function as both bioanode and biocathode (Hartline and Call, 2016). Acetate was used according to previous studies suggesting the enrichment of electro-active bacteria using acetate-fed medium (Logan et al., 2019), whereas the addition of butyrate could increase the diversity of the microorganisms in the anodic biofilm. 2 g/L COD was provided in the reactors for the first almost 30 days of the enrichment step, while it was changed to 1 g/L COD to provide the conditions similar to seawater before switching to bidirectional conditions on day 50. For enabling bidirectional biofilm activity, the organic compounds were removed from the media and 1 g/L NaHCO_3_ was added to the electrolytes to ensure the availability of sufficient inorganic carbon required for cathodic reactions.

As bacterial source, sediment samples were collected from the German coasts of the North Sea (coordinates 53°49'50.3"N 8°52'51.3"E) and the Baltic Sea (coordinates N 54° 18.205968 E 13° 43.078532) during September 2020. They were sampled, together with some overlying seawater, from a depth of *ca.* 20 cm from the sediment surface and *ca.* 30 cm from the seawater surface, and were kept in the fridge at 4°C before inoculating the reactors. Selected chemical sample characteristics are presented in the following table.

**Table:** Table - Characteristic of the sediment samples collected from North and Baltic Sea

Inoculum	pH	COD (mg/L)	Sulfate (mg/L)
North See sediment	7.6 ± 0.1	961.2 ± 9.1	520 ± 7
Baltic Sea sediment	7.4 ± 0.2	872.3 ± 5.2	454 ± 4

For inoculation, the sediment samples were shaken thoroughly and, after heavy mineral and particle components were allowed to settle, 20 ml of supernatant per reactor was sampled and supplemented into the working compartments of the bioelectrochemical reactors. The denomination “North biofilm” and “Baltic biofilm” thereby refers to the origin of the respective inoculum sources. The counter compartments contained 100 ml of the medium used in the working compartment without organics and inoculum. The pH of the medium in both compartments was 7.4 ± 0.1 after preparation and was measured regularly during the experiment. A triplicate of reactors was operated for each inoculum source. In addition, a sterile reactor was used as an abiotic control. To remove the oxygen in the electrolytes the anodic and cathodic medium were purged with N_2_/CO_2_ (80/20) for 30 minutes. The reactors were incubated at 33 ± 1°C and stirred at 180 rpm. To control the potential at the working electrodes, the reactors were connected to a potentiostat (VMP-3, BioLogic Instruments, France) and the desired potential was applied at the electrode (3 electrode configuration) while the current was monitored.

Primary biofilm cultivation was performed under heterotrophic conditions, i.e., at anodic potential and using organic carbon as substrate. For this purpose, a constant electrode potential of 0.2 V was applied using chronoamperometry (CA). The cultivation was performed in semi-batch mode, with replenishment of the medium after each batch cycle. The biofilm development was monitored via the generated anodic current and through regular cyclic voltammetry (CV), as well as electrochemical impedance spectroscopy (EIS). CVs were performed at the scan rate of 1 mV s^-1^ under turnover conditions (at peak current) and under non-turnover (at the end of the batch cycle) and the second scans were reported; Impedance measurements were performed under turnover conditions after reaching peak current. The amplitude of the sinusoidal excitation signal was set to 30 mV. The investigated frequencies lay between 1000 kHz and 100 mHz. Per decade, 30 frequency points were recorded, and each frequency was measured five times. The AC signal was superimposed with a constant DC potential of 0.2 V.

Based on a high similarity of CV and EIS data between the replicates, the figures presented in this article are exemplary figures representative for the respective biofilms.

For establishing and studying bidirectional (electrotrophic-electrogenic) biofilm behavior and current storage, the heterotrophic growth medium was replaced by a medium containing CO_2_ (from purging) and 1 g L^-1^ NaHCO_3_ as sole carbon sources. A periodic potential reversal was applied to establish bidirectional electron transfer. The potential reversal was carried out by changing the poised electrode potential from 0.2 V to -0.8 V every 2 hours. The transition between the potentials was achieved by a linear potential ramp (linear potential sweep, 1 mV s^-1^) in order to avoid physical stress to the microorganisms and to minimize double layer charging based capacitive currents. The combination of one anodic and one cathodic condition, including the transition between both potentials, was considered as one loop. The periodic potential reversal experiment was performed almost 40 hours over 10 loops. Cyclic voltammograms (scan rate 1mV s^-1^) were recorded regularly to study the electrochemical biofilm properties.

#### Chemical and microscopic analyses

To investigate the consumption of organic compounds during the anodic biofilm enrichment, or the production of organic compounds during cathodic biofilm operation, medium samples were regularly collected from the reactors and were analyzed using high performance liquid chromatography (1260 Infinity II chromatograph, Agilent Technologies, USA equipped with an Aminex HPX-87H column (Bio-Rad Laboratories, USA), a reference index detector and a diode array detector). The machine was calibrated to detect volatile fatty acids such as formate, acetate, propionate, butyrate, valerate and hexanoate, as well as methanol and ethanol in the samples. The gas composition in the reactor headspaces (H_2_, CO_2_ and CH_4_) was measured using gas chromatography (Thermo scientific, FOCUS GC) equipped with a thermal conductivity detector and a ShinCarbon ST Micropacked column (Restek, USA), using helium as a carrier gas.

Raman spectroscopy (InVia REFLEX, Renishaws, UK) equipped with a laser wavelength of 532 nm (Nd:YAG laser) and a Leica-Microscope DM 2700 at 20 fold magnification was used for speciation analysis of the biofilms (Beuth et al., 2020). In addition, electrode images were captured using scanning electron microscopy (SEM, Zeiss EVO LS 10). For SEM analysis, 1 cm x 1 cm samples of the electrodes were prepared by fixing in 2% paraformaldehyde (PFA) in aqueous phosphate buffer for 30 minutes (Esquivel et al., 2020). The fixed biofilms were then stored in a 70% ethanol solution at -20°C. Electrodes were dried and gold coated before SEM analysis.

#### Microbial community analysis

Microbial community analysis was performed from the bidirectional biofilms after terminating the reactors. Two samples, each 1 cm x 1 cm of the graphite felt electrode from each reactor, were cut and prepared for the community analysis. The species in the biofilms were identified by AMODIA Bioservice GmbH, Braunschweig, Germany. Briefly, total microbial DNA was isolated from the samples, and was used for a universal amplification of a 16S rDNA fragment for the detection of bacteria. The amplified PCR products were separated using the single strand conformation polymorphism (SSCP) method. SSCP analysis detected sequence variations (single-point mutations and other small-scale changes) through electrophoretic mobility differences. DNA that contained a sequence mutation (even a single base pair change) had a measurable mobility difference when subjected to non-denaturing (or partially denaturing) conditions. For every sample, a bacterial DNA profile was generated. Each band in the profile represented a specific DNA sequence and was assigned to a bacterial species. For species identification, the bands were isolated and analyzed using DNA sequencing and database inquiry. The alignment with characterized sequences in the database allowed the identification of the next homologous sequence and the respective bacterial species. Furthermore, selected sequences were aligned using “msa” package (Bodenhofer et al., 2015) and phylogenic tree was plotted using “ape” package (Paradis et al., 2004, 2019) in R.

### Quantification and statistical analysis

The data in this manuscript are shown as the means ± SD.

## Footnotes

Supplemental information can be found online at https://doi.org/10.1016/j.isci.2021.102822.
