# Supplementary material for: Bidirectional electroactive microbial biofilms and the role of biogenic sulfur in charge storage and release
Source: iScience. 2021 Jul 7;24(8):102822. doi: 10.1016/j.isci.2021.102822 (PMC8313490; doi:10.1016/j.isci.2021.102822)
Supplement: Document S1. Figures S1–S16 [file mmc1.pdf]

iScience, Volume *24*

Supplemental information

Bidirectional electroactive microbial biofilms and the role of biogenic sulfur in charge storage and release

Paniz Izadi, Marten Niklas Gey, Nicolas Schlüter, and Uwe Schröder

Supplementary

A

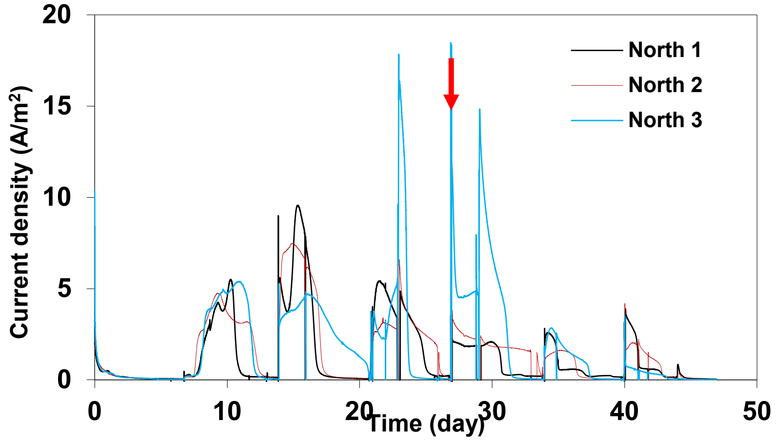

B

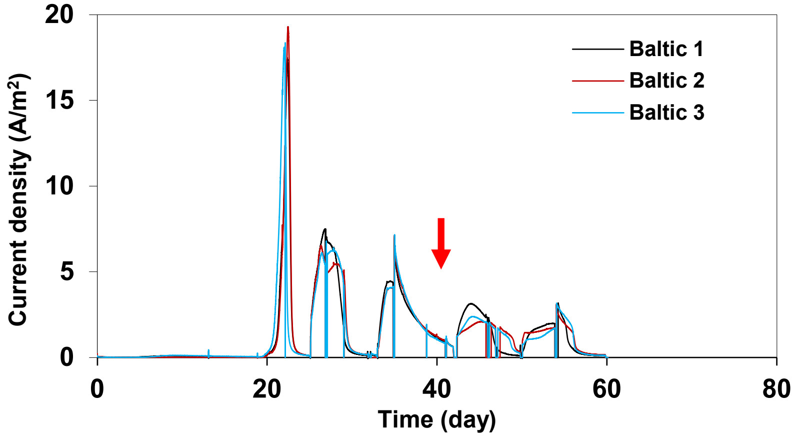

Figure S1. Electricity generation in A) North and B) Baltic reactors during the anodic enrichment, related to Figure 1.

The red arrows indicate when total substrate concentration decreased from 2 g L^{-1} COD to 1 g L^{-1} COD.

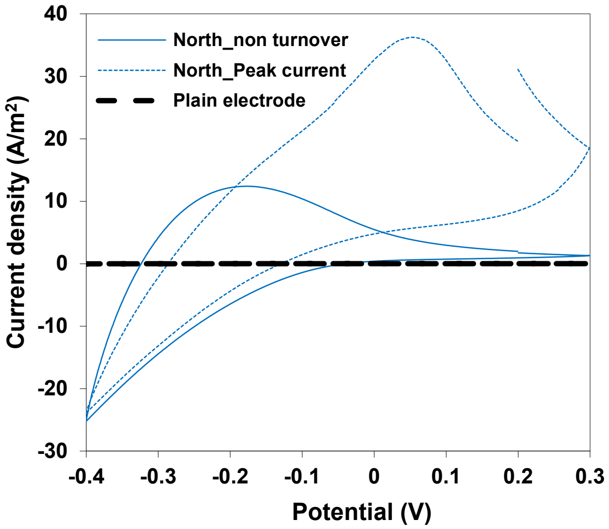

Figure S2. Cyclic voltammetry performed at a North-biofilm after development of anodic biofilm, related to Figure 1

The voltammograms were recorded at the peak current and under non-turnover conditions compared with the plain electrode (Scan rate = 1 mV s^{-1}).

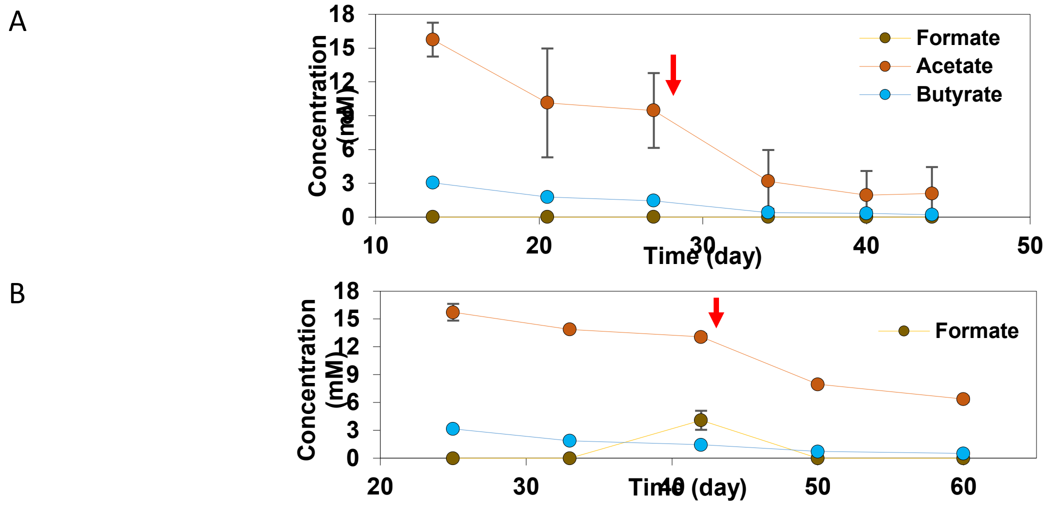

Figure S3. Quantitative analysis (absolute concentrations) of the substrate consumption during anodic cultivation, related to Figure 1

Absolute concentrations of formate, acetate and butyrate in A) North and B) Baltic reactors at the end of each batch cycle. The red arrows indicate when total substrate concentration decreased from 2 g L^{-1} COD to 1 g L^{-1} COD.

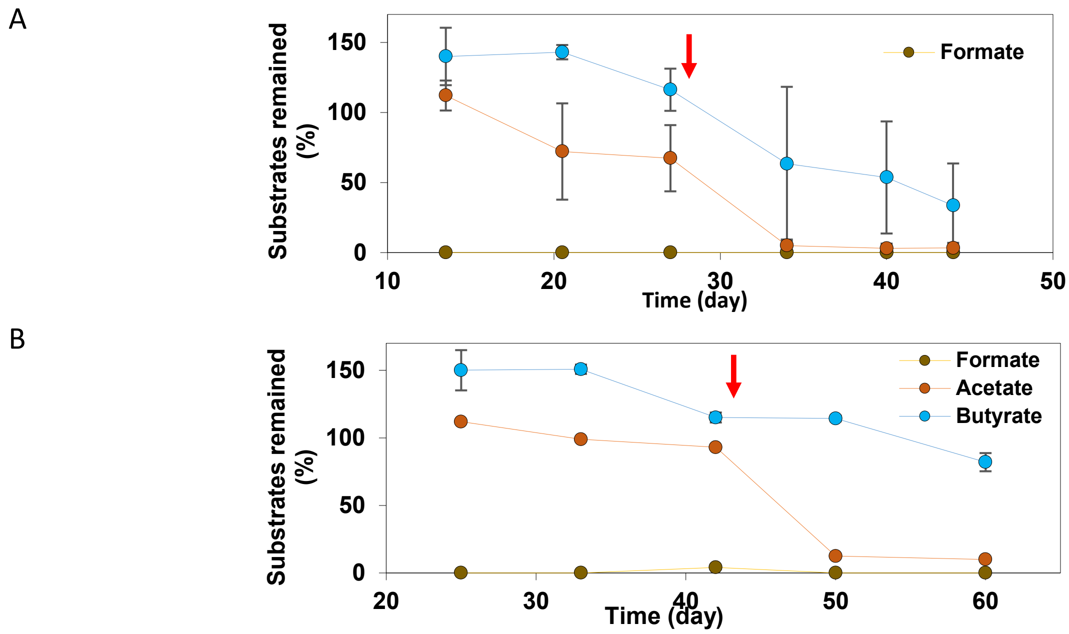

Figure S4. Quantitative analysis (relative concentrations) of the substrate consumption during anodic cultivation, related to Figure 1

Relative concentrations of formate, acetate and butyrate remained in anolytes of A) North and B) Baltic reactors at the end of each batch cycle. The red arrows indicate when total substrate concentration decreased from 2 g L^{-1} COD to 1 g L^{-1} COD.

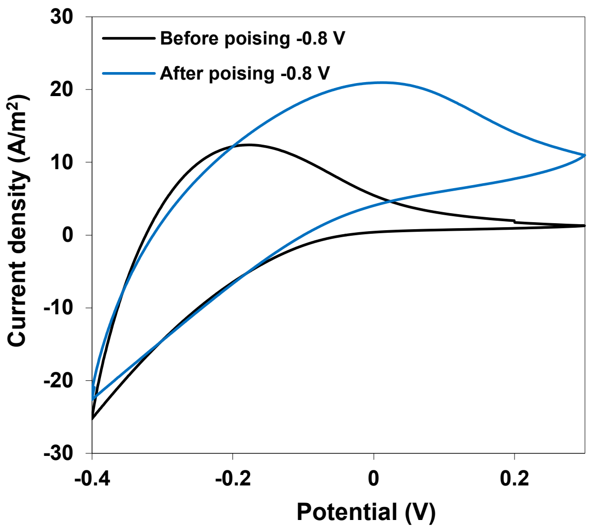

Figure S5. Impact of a cathodic polarization on the non-turnover biofilm voltammetry, related to Figure 2

The figure shows a cyclic voltammogram of a North-biofilm under non-turnover conditions, before and after polarization at a potential of -0.8V for one hour. (Scan rate 1 mV s^{-1}).

A

B

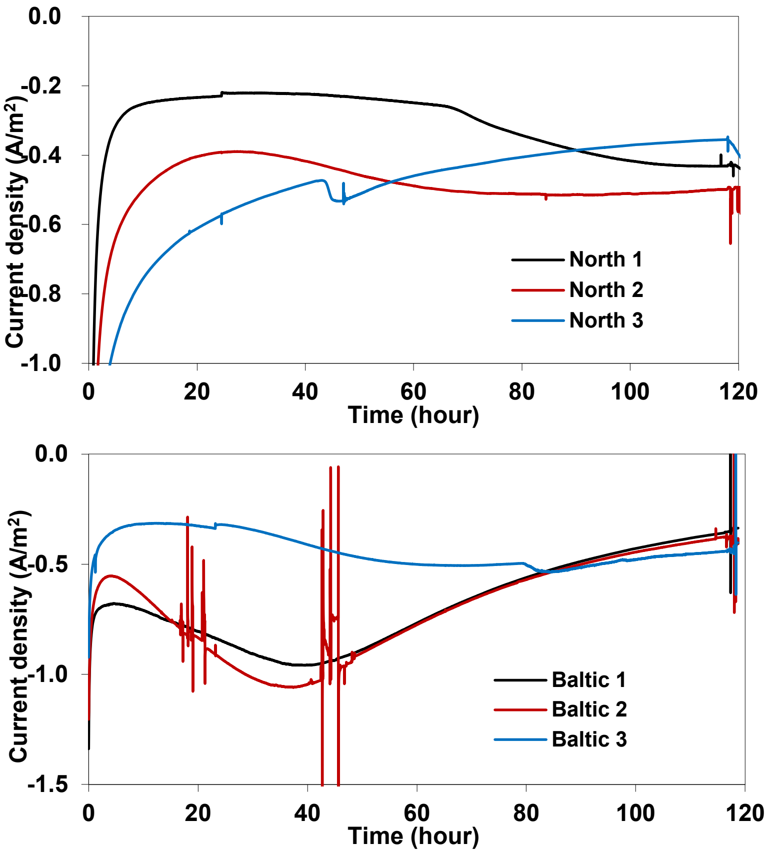

Figure S6. Long-term cathodic current generation, related to Figure 2

A) North and B) Baltic biofilms; the experiments were recorded over 5 days; the applied potential was -0.8 V.

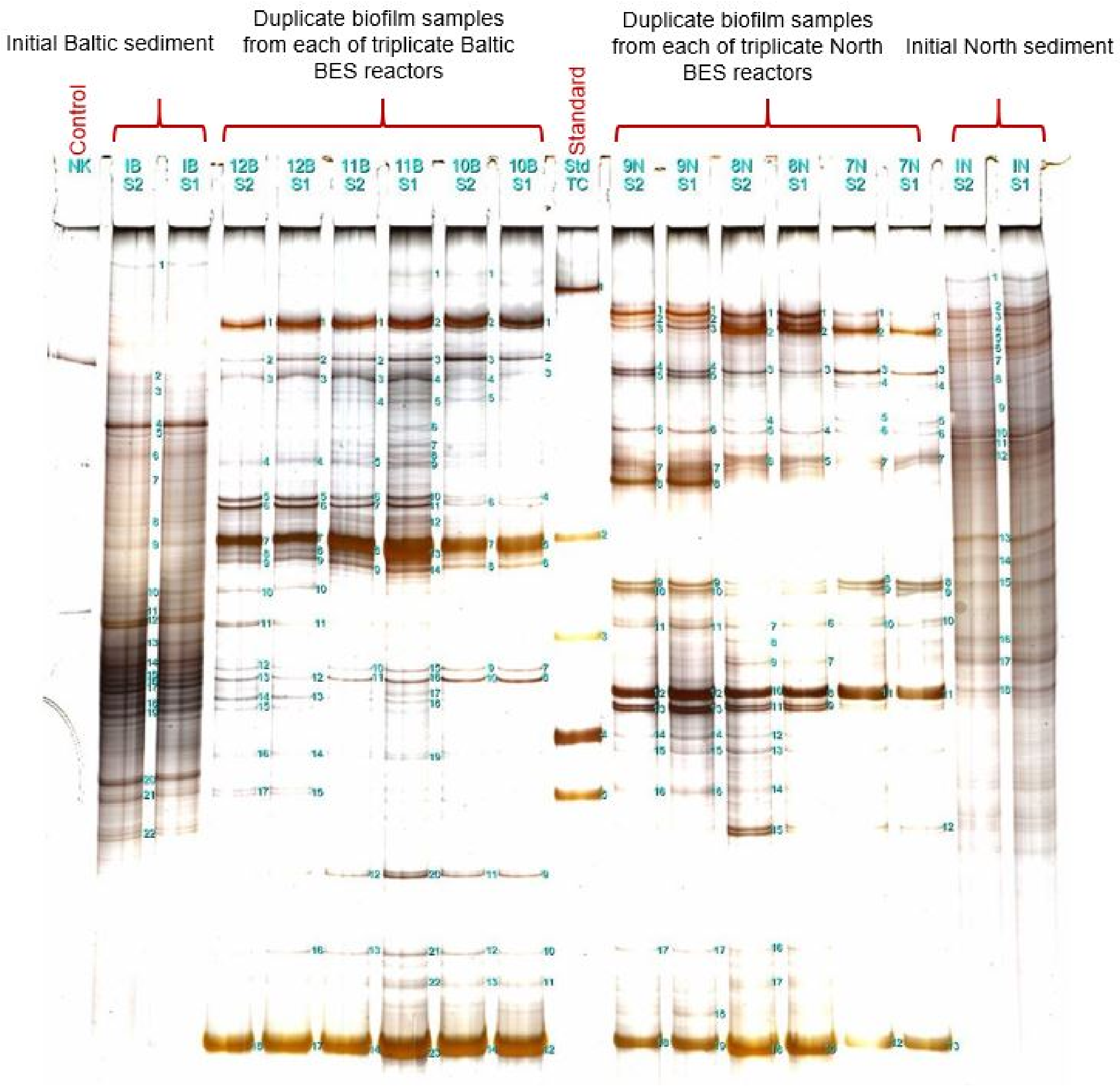

Figure S7. DNA gel images, related to Figure 5

The gels were imaged from the initial inoculum sources of Baltic and North sediments, biofilms of the Baltic and North reactors, negative control and standard sample.

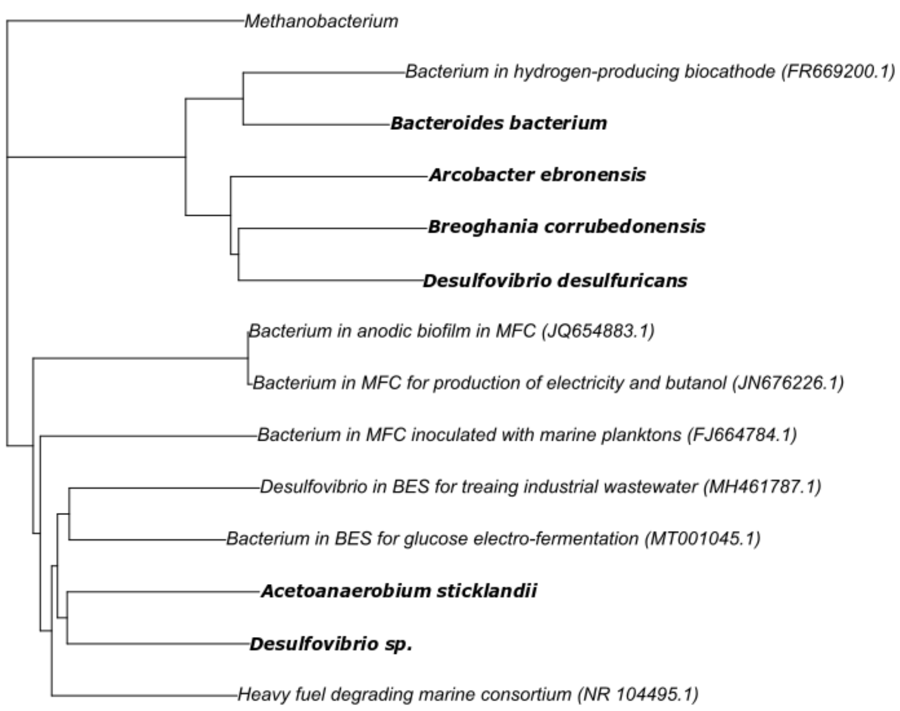

Figure S8. Phylogenetic tree of the 16S rRNA gene sequences, related to Figure 5

The phylogenetic tree was extracted from the community analysis of the biofilms developed in North and Baltic reactors, compared with highly similar sequences extracted from NCBI Nucleotide database collection. *Methanobacterium* was used as an outgroup from Archaea.

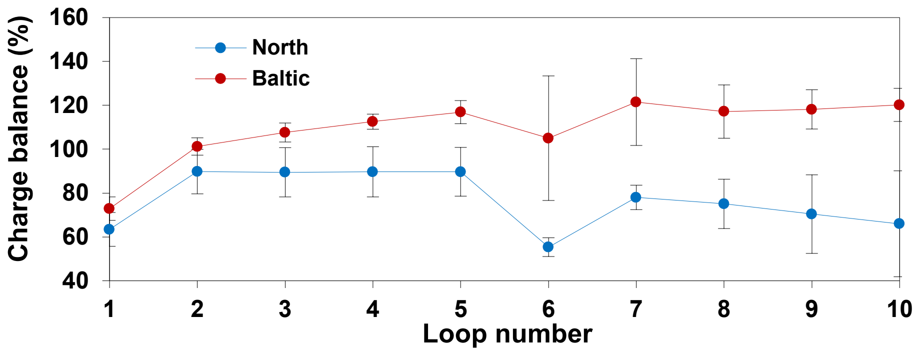

Figure S9. Cumulative charge and charge balance over 10 loops of periodic potential conversion in North and Baltic reactors compared to plain electrode, related to Figure 3

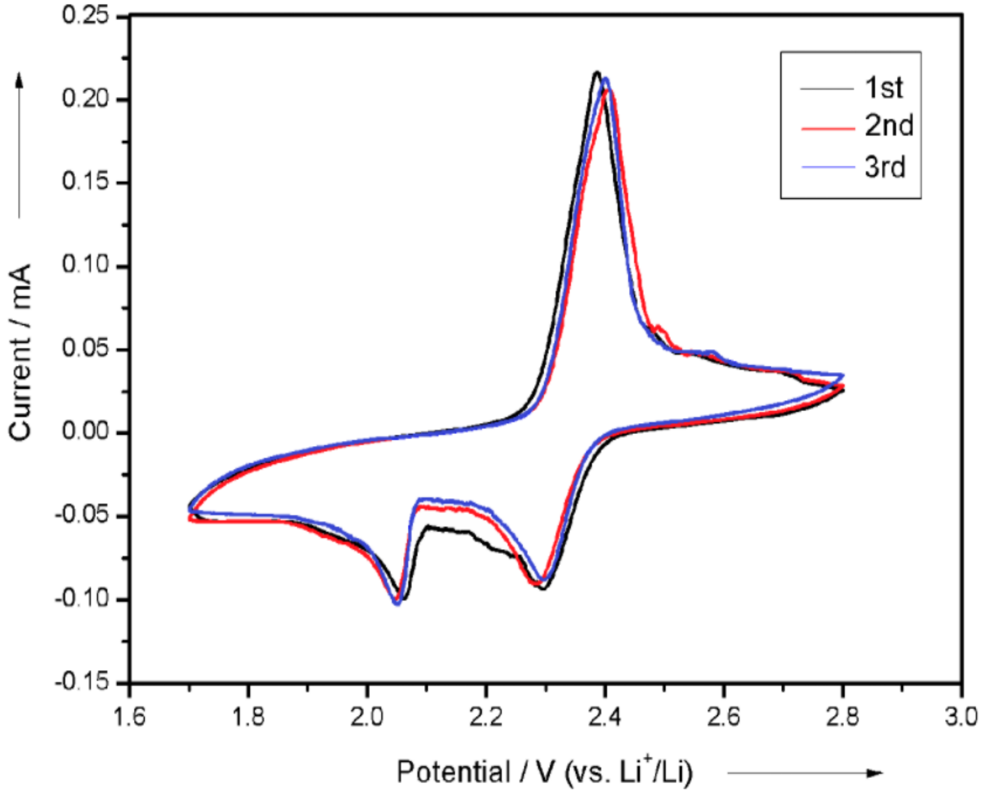

Figure S10. Cyclic voltammetry of a sulfur cathode of a Li-S battery, related to Figure 6

Figure with permission from Wang and Zhang, 2020.

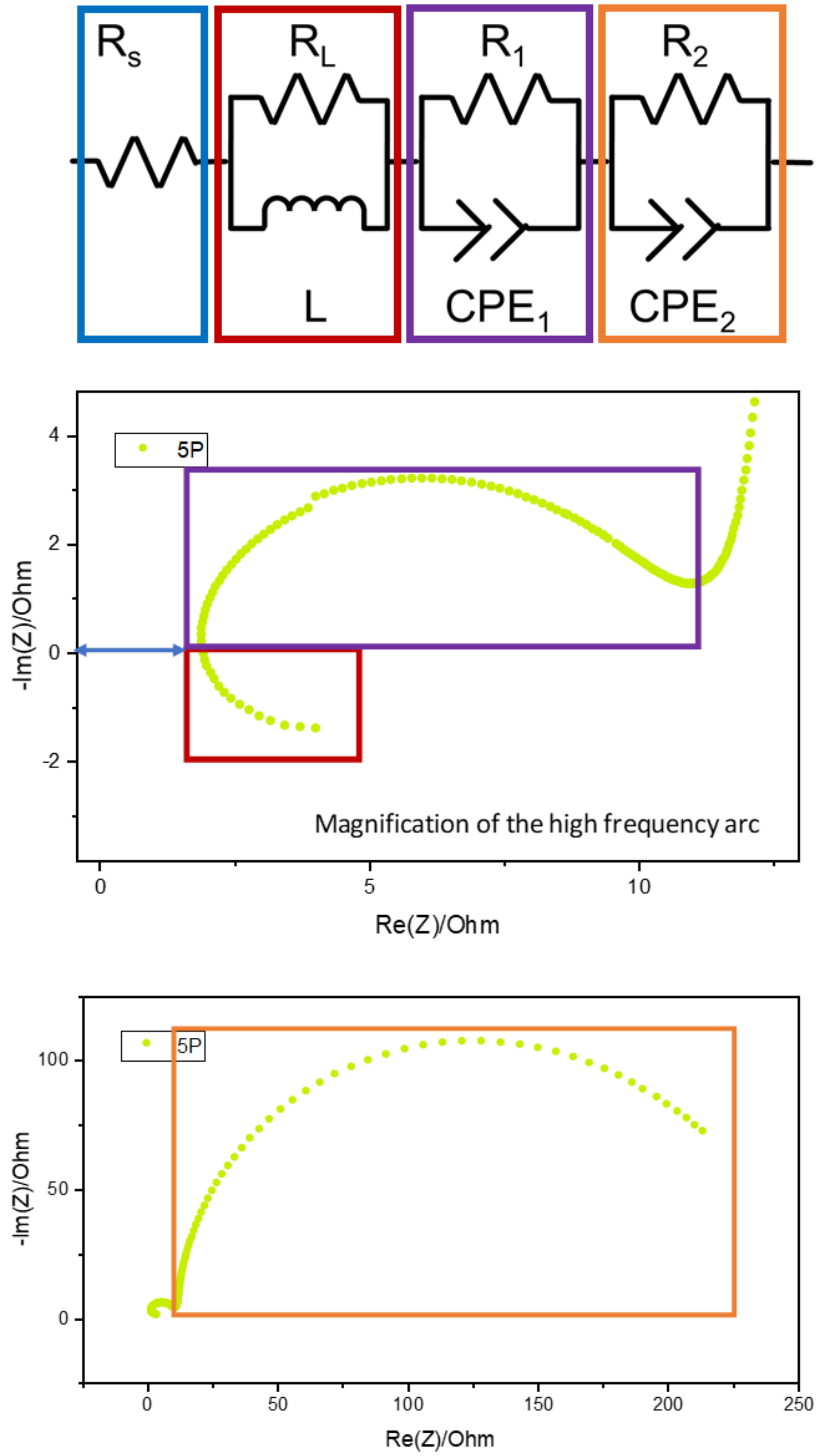

Figure S11. Representation of the fitting / the assignment of equivalent circuit elements, related to Figure 8

A CPE element was used for the equivalent circuit fitting, since pure capacitance cannot be measured directly. Instead, the CPE-values "*T*" and "*P*" are obtained, from which the capacitance can be derived based on the equation:

$$C = \frac{(R * T)^{1/P}}{R}$$

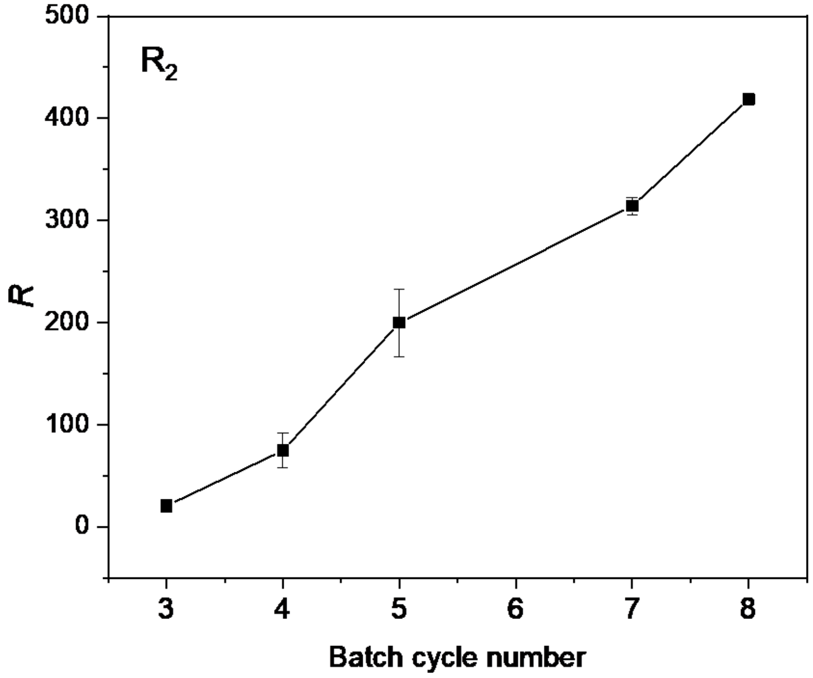

Figure S12. Development of the charge transfer resistance R_2 as derived for the North biofilm electrodes, related to Figure 8

The values are based on the biofilms from reactors 1 and 2. Reactor 3 was discarded due to a strong outlier in cycle 8.

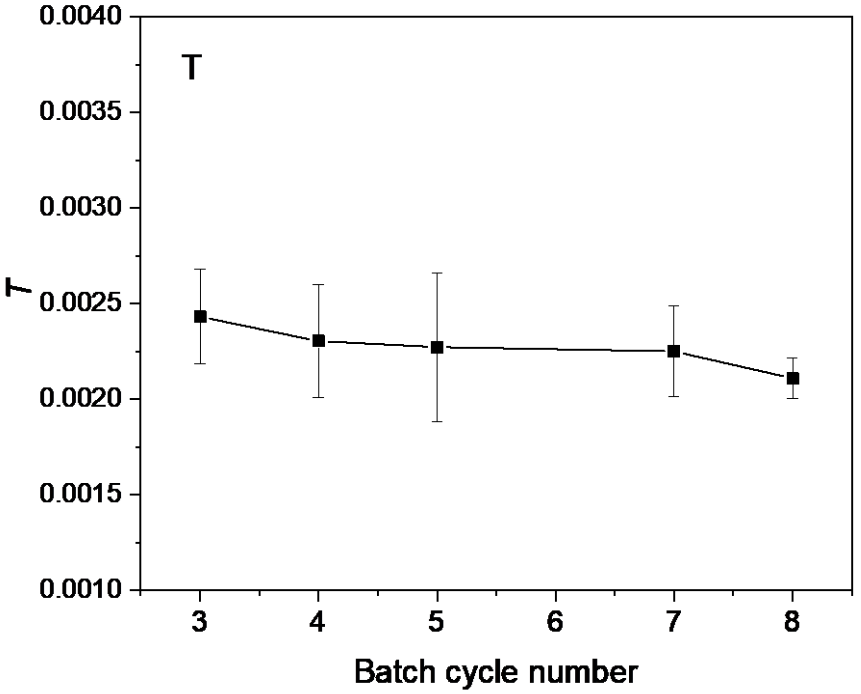

Figure S13. Development of the CPE factor T for the North biofilm electrodes of the reactors 1-3, related to Figure 8

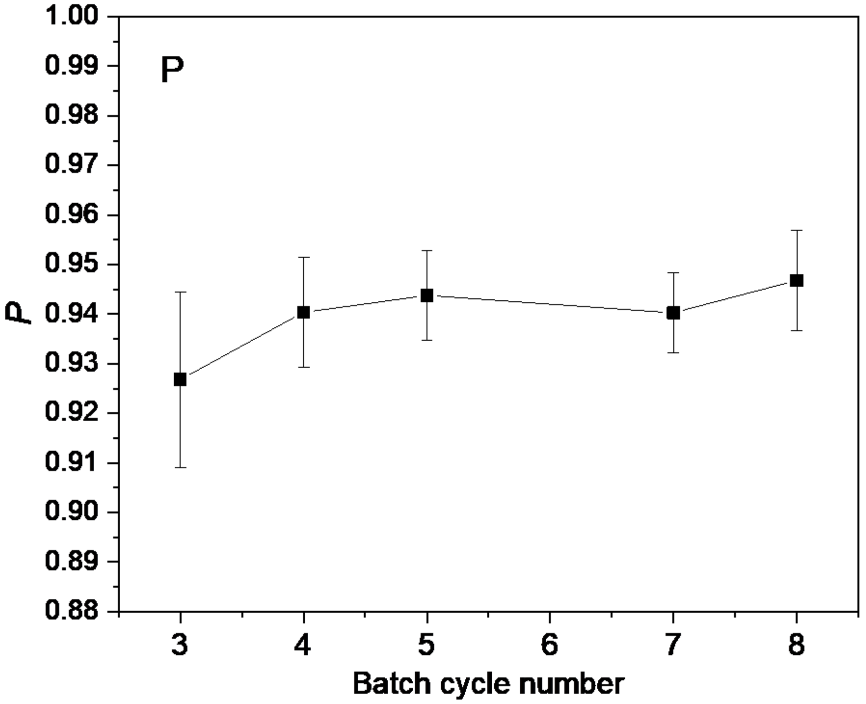

Figure S14. Development of the CPE factor P for the North biofilm electrodes of the reactors 1-3, related to Figure 8

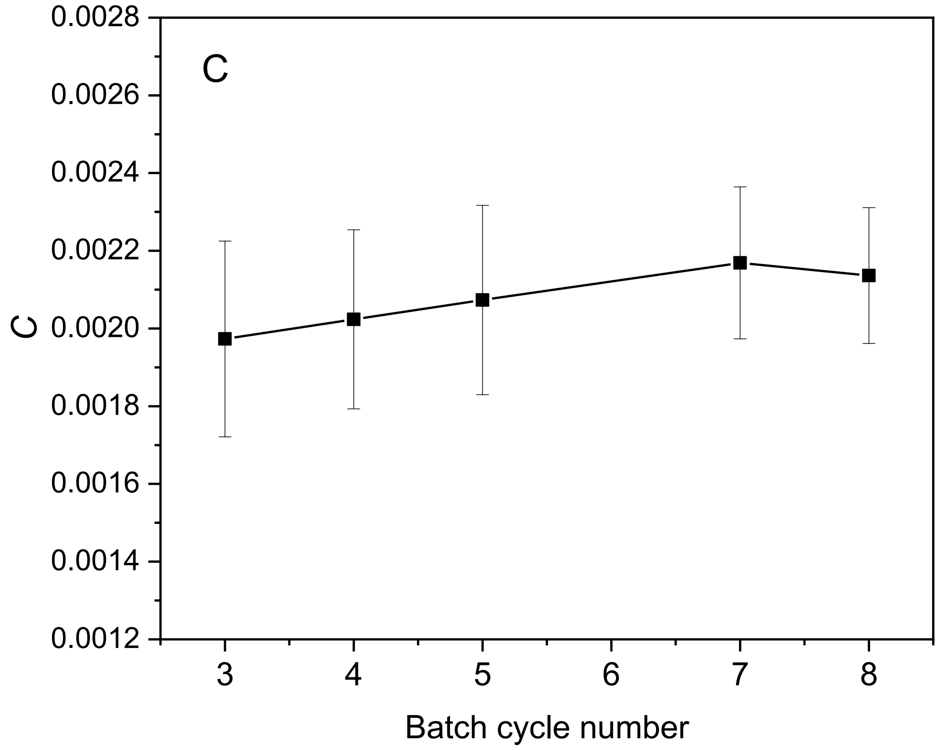

Figure S15. Development of the Capacitance of the North biofilm electrodes of the reactors 1-3, related to Figure 8

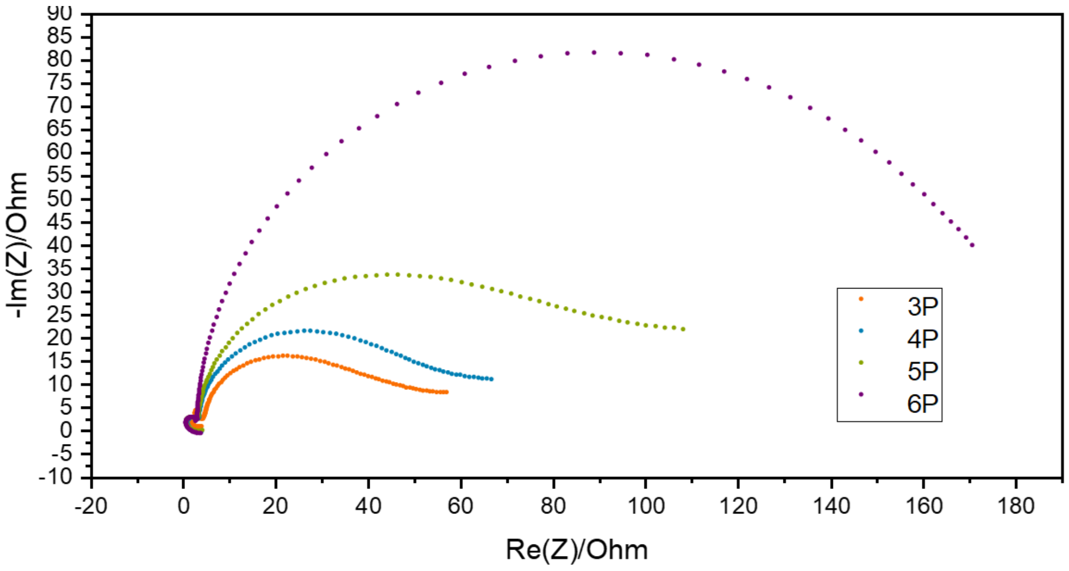

Figure S16. Exemplary impedance spectra (Nyquist plots) of a Baltic-biofilm electrode during the course of the anodic cultivation, related to Figure 8

The spectra are based on the biofilm from reactor 2 and were recorded at the respective current maxima of the cultivation semi-batch cycles, at a polarization potential of +0.2V.
